# Undoped and Nickel-Doped Zinc Oxide Thin Films Deposited by Dip Coating and Ultrasonic Spray Pyrolysis Methods for Propane and Carbon Monoxide Sensing Applications

**DOI:** 10.3390/s20236879

**Published:** 2020-12-01

**Authors:** Tangirala Venkata Krishna Karthik, María de la Luz Olvera, Arturo Maldonado, Rajesh Roshan Biswal, Heberto Gómez-Pozos

**Affiliations:** 1 Escuela Superior de Tepeji del Río, Ingeniería Industrial, Universidad Autónoma del Estado de Hidalgo, Avenida del Maestro No. 41 Colonia Noxtongo, Segunda Sección, 42855 Tepeji del Río, Hidalgo, Mexico; enkata_tangirala@uaeh.edu.mx; 2 SEES, Departamento de Ingeniería Eléctrica, Centro de Investigación y de Estudios Avanzados del Instituto Politécnico Nacional, CINVESTAV-IPN, 07000 Mexico City, Mexico; molvera@cinvestav.mx (M.d.l.L.O.); amaldo@cinvestav.mx (A.M.); 3 Escuela de Ingeniería y Ciencias, Tecnológico de Monterrey, Avenida General Ramón Corona 2514 Nuevo México, 45138 Zapopan, Jalisco, Mexico; rroshanb@tec.mx; 4 Área académica de computación y electrónica, Universidad Autónoma del Estado de Hidalgo, 56092 Pachuca, Hidalgo, Mexico

**Keywords:** zinc oxide, propane gas, dip coating, ultrasonic spray pyrolysis

## Abstract

Undoped and nickel-doped zinc oxide thin films were deposited on sodalime glass substrates by utilizing dip coating and ultrasonic spray pyrolysis deposition techniques. In both cases zinc acetate and nickel acetylacetonate were used as zinc precursor and nickel dopant source, respectively. XRD analysis confirms the ZnO wurtzite structure with (002) as the preferential orientation.SEM studies show the formation of two types of morphologies, primarily a porous spherical grains with a grain size distribution from 40 to 150 nm and another, rose-like structures with size distribution from 30 to 200 nm, based on different deposition techniques utilized. The elemental depth profiles across the films were investigated by the secondary-ion mass spectrometry (SIMS). Different gas sensing responses of all ZnO films were obtained for both propane and carbon monoxide gases, at different gas concentrations and operating temperatures. The highest sensing response (~6) for undoped ZnO films was obtained for films deposited by ultrasonic spray pyrolysis (USP). Nevertheless, the highest sensing response (~4 × 10^4^) for doped ZnO films was obtained for films deposited by dip coating method. The behavior of sensing responses is explained in detail based on the morphological properties and the amount of Ni impurities incorporated into the crystal lattice.

## 1. Introduction

Zinc oxide (ZnO) thin films are well-known as one of the most promising materials because of their low dielectric constant [1], high electromechanical stability [2], high direct wide band gap (~3.3 eV) [3]. In addition to low cost and high abundance on earth, ZnO hexagonal wurtzite crystal structure [4] possess advantages like high optical transmittance (80–90%) [5], abundant surface oxygen vacancies, ecofriendly [6]. In fact, ZnO thin films have encountered a wide range of applications in electrical and opto-electronic devices, such as varistors, thin film transistors, transparent contacts in solar cells, pressure sensors and gas sensors, among others [7,8,9]. Human activity has had a significant impact in the environment, which is reflected mainly in the air pollution, due to the combustion of different products derived from hydrocarbons. Among the derivatives of the hydrocarbons, we can obtain propane, which is economic and widely used in the industry, as it is in domestic environments. Moreover, propane (C_3_H_8_) competes with other sources of energy such as butane and is used mainly in heating domestic environments and in a lesser way by stoves and ovens. It is considered that the legal airborne permissible exposure limit is 1000 ppm over an 8–10 h work shift at an auto-ignition temperature of 435 °C, which is the reason it is inevitable to have gas sensing devices that are able to constantly monitor gas concentrations below this limit [10,11,12]. Moreover, part of the CO gas is produced from burning fuels like kerosene, petroleum, carbon, wood, gasoline, natural gas and propane, with an auto-ignition temperature of 625 °C. It is considered that even at small concentrations of 70 ppm for a relatively short exposition time is detrimental to health [13,14,15]. The propane and carbon monoxide sensing have been analyzed by different semiconducting oxides such as MgSb_2_O_6_ [16], TiO_2_ [17], CdS, [18], SnO_2_ [19] and ZnO [20], deposited by different deposition techniques and conditions. We have found that CO and C_3_H_8_ can be detected above the 100 °C operating temperature, with sensing responses varying between 0.1 to 4000 at 200–400 ppm of propane, and the response and recovery time within a range of 1 to 5 s [21,22]. In continuation, a comparative table between the best results obtained in this work and those of reference [21,22], is shown in Table 1. It can be seen that, our results are competitive and, in some cases, much superior to others, as in the case of ZnO films deposited by dip coating.

Different physical and chemical methods [23,24,25,26,27,28,29,30,31,32,33] have been used previously by different authors to deposit high quality ZnO thin films under optimum deposition conditions in order to obtain higher gas sensing responses. Chemical methods such as dip coating and ultrasonic spray pyrolysis, (USP) present a wide range of advantages, such as inexpensive equipment (non-vacuum method), high throughput, facile synthesis, simple processing, and bulk and uniform deposition of films, both of which produce ZnO films with different physical characteristics according to their nature of synthesis [34,35,36,37]. Based on this, the interest of this work is to deposit ZnO thin films by two chemical deposition methods, namely, dip coating and USP, using the same starting solution Zn acetate and doping solution nickel acetylacetonate. This way, a comparative analysis is done to obtain results of the ZnO films deposited by both deposition techniques and the effect of its physical properties on the sensing responses is analyzed. Morphological properties and the change in doping concentration are discussed to a greater extent because these are some of the factors that affect directly the sensors performance as gas sensors.

## 2. Experimental Procedure

### 2.1. ZnO Preparation Process

Detailed experimental procedure followed in this work is explained in the upcoming sections. Although there exists a variety of technique to deposit ZnO films [38,39,40], in this work thin films were deposited by ultrasonic spray pyrolysis, USP and sol gel dip coating. ZnO films were deposited on soda-lime glass (sizes of 2.5 cm × 5 cm) substrates. The procedure to clean the substrates is described in [41]. Deposition process of ZnO films utilizing sol-gel dip coating and USP methods were as follows.

#### 2.1.1. Films Deposited by Sol-gel Dip Coating

**Figure 1:**
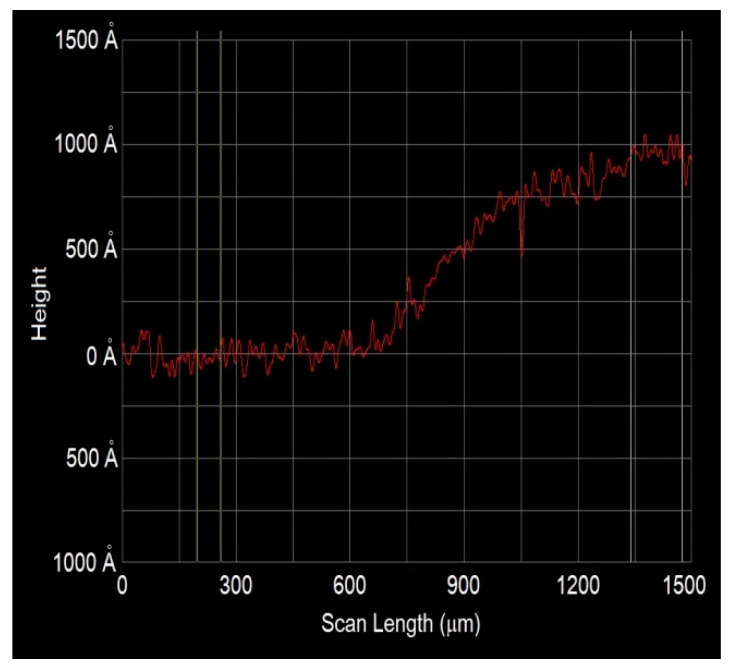
Step measured by a talkstep for a undoped ZnO film deposited by dip coating.

Undoped ZnO films were deposited by the sol-gel dip coating method, the solution was prepared by dissolving zinc acetate dehydrate (Zn(CH_3_COO)_2_∙2H_2_O, Sigma Aldrich, United State) in a homogeneous mixture of 2-methoxyethanol (Methyl glycol, CH_3_OCH_2_CH_2_OH, Sigma Aldrich, United State) and monoethanolamine (MEA, NH_2_CH_2_CH_2_OH, Sigma Aldrich, United State) to a ratio of 1:1 and 0.6 Molar concentration. The deposition was realized by dipping the glass substrate several times, primarily 6 dippings in the starting solution were used to obtain undoped ZnO and later 2, 4, 6 and 8 dippings in the doping solution were performed for obtaining Ni doped ZnO films. The doping solution was prepared from nickel acetylacetonate dissolved in deionized water and the doping concentration was obtained by varying the amount of dippings or immersions in this solution. However, the sensing process is a superficial process, it was found that 6 immersions in the starting solution are enough to ensure the formation of ZnO, and 2, 4, 6 and 8 dippings in doping solution are made to analyze the Ni concentration effect on the sensing properties. An annealing process in air and at 200 °C was carried out for 10 min after each dipping to dry and remove the residual solvents. To improve the film homogeneity, a final annealing process was performed in air, at 450 °C and 1 h. The average thickness of films for 6 dippings resulted in ~ 100 nm for all films. To check the thickness of a ZnO film with 6 dippings, a step measured by a talkstep is shown in the Figure 1. Lastly, undoped and doped ZnO films were compared to analyze the effect of Ni on the sensing properties.

#### 2.1.2. Films Deposited by Ultrasonic Spray Pyrolysis

The starting solution was prepared by dissolvingzinc acetate in a mix of acetic acid (CH_3_CO_2_H, Sigma Aldrich, United State), deionized water and methanol (Methyl alcohol, CH_3_OH, Sigma Aldrich, United State), (50:50:900) volume proportions, respectively, with a 0.2 Molar concentration of Nickel acetylacetonate (Nickel(II) acetylacetonate, Ni(C_5_H_7_O_2_)_2_ Sigma Aldrich, United State) was the doping precursor added at 3 and 5 at. % with respect to Zn content. The final solution was stirred until a homogenous solution was obtained. Two substrate temperatures were selected, namely 400 and 450 °C (these substrate temperatures ensure the correct formation of the films) at a fixed1 ml/min solution spray rate. The films thickness was maintained constant by 5 min deposition spray time, resulting in a film thickness of approximately 160 nm for both the undoped and doped ZnO thin films (therefore the addition of the doping solution in the starting solution does not affect the film thickness). The film thickness of the doped ZnO films deposited by spray pyrolysis were kept similar to that deposited by dip coating, since higher sensing responses were observed at this thickness (160 nm).

### 2.2. Films Characterization

The structural properties of ZnO thin films were investigated by X-ray diffraction with Cu-Kα_1_ (λ = 1.5418 Å) radiation, with an angle ranging between 20 and 80°. A profilometer KLA-Tencor P-15 was used to measure the thicknesses and rms roughness of the samples deposited (measurements can be achieved with up to a 0.5 Å resolution). Scanning Electron Microscopy (SEM) JEOL JSM-5900LV was employed to study the surface topography of the films, the electron high tension (EHT) was 1 kV, some other data, aperture size = 20 µm, WD = 3.6 mm and magnification = 80 KX and SIMS measurements were performed to compare the Zn, O and Ni concentration profile in the ZnO films. The equipment was a TOF-SIMS-5 secondary ion mass spectrometer from ION-TOF GmbH company. A double beam analysis regime was used to measurements: a focused Cs+ ion beam with energy of 500 eV and ion current of 60 nA, the area of raster scanned was 500 × 500 μm^2^ and a pulsed Bi3+ ion beam was used to analyze a 150 × 150 μm^2^ in a central area of the sputtered crater. Secondary CsM+ cluster ions (where M is an element of interest) were monitored to minimize the SIMS matrix effect at the semiconductor interface and experimental crater depth was measured by a Dektak XT profiler for recalculation of depth profile.

**Figure 2:**
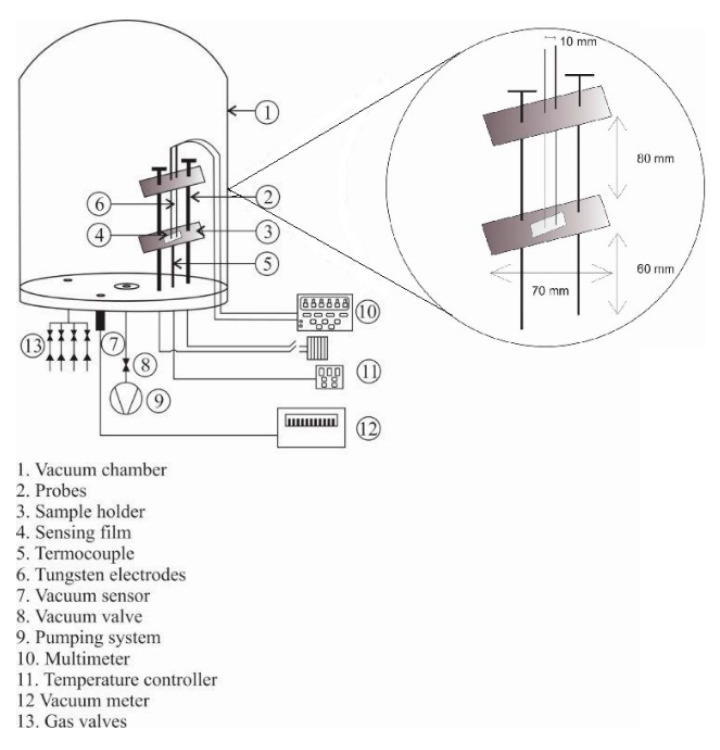
Schematic diagram of the system used to measure sensing properties in controlled atmospheres and temperatures.

In order to investigate the sensor response (S), surface electrical resistance measurements in air and in different concentrations of propane gas (ΔR) were obtained, utilizing a Keithley 2001 digital multimeter with 1 GΩ range, 100 Ω resolution and 4.4 nA current source. The surface electrical resistance was measured by a two-point probe method in which, two linear ohmic contacts (checked by I-V curve tracers) separated by 1 cm, were deposited onto the films by employing silver paint, the contacts were made of nichrome wire 1 mm in diameter and 10 cm tall. Surface electrical resistance measurements were recorded manually. Each sample was placed into a chamber with the following parameters: minimum obtained base pressure of 0.61 × 10^−3^ atm, (humidity does not affect in the sensing measurements), chamber volume of 12,800 cm^3^, propane temperature 298 K (not affecting the temperature of the sample). Now from the parameters of the chamber as shown in Figure 2 and from the ideal gas equation, PV = nKT, the propane concentration in parts per million, i.e., ppm, is obtained as follows, first from ideal gas constant used, k is 82.05 cm^3^ atm/Kmol and the chamber parameters, the air molar concentration, n_air_, is obtained, by increasing the pressure in the chamber with propane, the molar concentration n_total_ is obtained, which includes the air and C_3_H_8_ concentration; therefore, the C_3_H_8_ molar concentration nC_3_H_8_ is obtained from the difference of n_total_ and n_air_, finally the ppm are obtained from the definition ppm = solute/solvent, where solute is C_3_H_8_ concentration in milligrams and solvent is chamber volume in liters. The measurement chamber allows the introduction of propane gas (C_3_H_8_, Praxair, 1000 ppm, balance nitrogen) in different concentrations starting from 1 to 500 ppm, and for carbon monoxide gas (CO Praxair, 1000 ppm, balance nitrogen) in concentrations from 1 to 300 ppm. The ambient gas under consideration was zero-grade air. For controlling the partial pressure in the chamber, a Leybold thermovac TM 20 controller with a gauge type Piranni connected was used, having an error range of less than 15%. Theparts per million of a gas is achieved by adjusting the pressure of the gas inside the chamber through a control valve that connects the gas tank and the chamber, leaving the valve open for the necessary time and once the pressure is achieved of any desired gas the valve is closed. The Table 2 shows the gas pressure values and their corresponding ppm in the chamber.

To achieve a uniform gas pressure throughout the chamber (since it is large, 10 liters), sometime is allowed to pass after the desired pressure is achieved (15 min) and thus a good gas distribution is achieved inside the chamber. The sensing response was measured from 100 to 300 °C, which was achieved by the usage of a cromel–alumel thermocouple, 2-prong configuration. Schematic diagram of the characterization system used for measurements shown in Figure 2.

Sensor response (*S*) is expressed as the ratio of the change in the surface electrical conductance in the presence of gas *G_G_* and with respect to air *G_O_* (where *G* = 1/ surface electrical resistance), according to the Equation (1):

(1) $$\mathrm{Sensor}\;\mathrm{response}\;\left(s\right)=\frac{G_{G}-G_{O}}{G_{O}}$$

## 3. Results and Discussion

The undoped ZnO films prepared by dip coating with six dippings in the starting solution was labeled as “ZnO, 6” while for Ni-doped ZnO films with two, four, six and eight dippings in the doping solution were labeled as, “ZnO:Ni, 6:2”, “ZnO:Ni, 6:4”, “ZnO:Ni, 6:6”, and “ZnO:Ni, 6:8”, respectively. For undoped ZnO films deposited by USP, two substrate temperatures were used, 400 and 450 °C and were labeled as “ZnO, 400 °C” and “ZnO, 450 °C” respectively. In the case of Ni doped ZnO films, these were deposited with two doping solutions, 3 and 5 at. % of Ni solutions with two substrate temperature of 400 and 450 °C. These films were identified as “ZnO:Ni, 3%, 400 °C”, “ZnO:Ni, 3%, 450 °C”, “ZnO:Ni, 5%, 400 °C” and “ZnO:Ni, 5%, 450 °C”.

### 3.1. Sensing Mechanism

The sensing mechanism in a type n polycrystalline material such as the one used in this work is described below: On the surface of the semiconductor, the atoms are interrupted in their orderly formation, leaving loose bonds with them, these bonds have to be occupied normally by oxygen by means of the trapping of a free electron, leaving the oxygen ionized negatively and the surface depleted of electrons, positively charged. In general, as the Fermi level moves from the lower part of the conduction band towards the center of the forbidden band, causing both the valence band and the conduction band to bend at higher energies, thus forming a potential barrier qVs [42,43]. The height of which depends on several parameters, as described in the following equation:

(2) $${\mathrm{qV}}_{s}=\frac{q^{2}N_{ss}^{2}}{2N_{D}}$$

where *q* is the charge of the electron, *N_ss_* is the density of surface traps, *ε* is the permitivity of the semiconductor and *N_D_* is the concentration of donors. Therefore, the surface conditions are vital for the sensing process, the higher the density of surface states, the greater the potential barrier, which can be achieved by placing more impurities on the surface of the semiconductor, and which in turn depends on the deposition technique used. The variation of this potential barrier depends on the dynamics of interaction of these surface states with the surrounding gas. It is preferable to have large potential barriers since their magnitude can vary more than a small barrier, and in this way increase the sensing response. However, high sensing response can also be achieved by having surfaces with a high effective surface area (ESA), due to the availability of a greater sensing area, which can be achieved by having a large amount of small grains.

The chemical reactions of CO and C_3_H_8_ on the surface are shown, wherein, the interaction of an CO molecule with a surface oxygen ion (O^−^) released a trapped electron, thus decreasing the electrical resistance on surface, as described in Equation (3):

(3) $${\mathrm{CO}}_{\mathrm{gas}}+\frac{1}{2}O_{2\mathrm{ads}}^{-}\to CO_{2\mathrm{gas}}+e^{-}$$

while Equations (4) and (5) describes the case of C_3_H_8_ with adsorbed oxygen:

(4) $$C_{3}H_{8}+2O_{\mathrm{ads}}^{-}\to H_{2}O+C_{3}H_{6}:O+e^{-}$$

(5) $$C_{3}H_{6}:O+8O_{\mathrm{ads}}^{-}=3CO_{2}+3H_{2}O+e^{-}$$

where $O_{\mathrm{ads}}^{-}$ and $O_{2\mathrm{ads}}^{-}\;$is the adsorbed oxygen and molecular oxygen, respectively. C_3_H_6_:O is the result of breakage of a propane molecule bonded to adsorbed oxygen.

ZnO film has also been manufactured as a reliable gas sensor for a long time [44,45,46,47,48,49,50,51,52,53,54], the addition of cationic dopants such as Cu, Ru, Cr or Ni, into the ZnO lattice, increases the sensing response due to the creation of surface states [55]. As a matter of fact, an enhancement in the sensing detection has been reported for hydrocarbons C_n_H_2n+2_ [56,57,58]. In this work, we used Ni^2+^ as an impurifier since its ionic radius is 0.69, somehow smaller than the ionic radius of Zn, i.e., 0.74 Å, not distorting the ZnO lattice crystal, and occupying mainly its substitute places of Zn^2+^, since it can donate 2 electrons to the oxygen, just as Zn does, (at doping solution 5 at. %, the maximum solubility of Ni in ZnO is reached, occupying the Ni interstitials places [59]. Furthermore, modulation of the surface morphology, increasing the amount of grain boundaries, yields an increase in the sensing response. Although there have been many works reporting the properties of ZnO as a gas sensor, not many works have been devoted to a complete understanding of the phenomena taking place on the film surface. It is found that varying Ni concentration in the ZnO films both surface morphology and structural properties are affected, and in turn affects the sensing responses; therefore, the Ni dopant increased the sensing response [60].

### 3.2. Structural Properties

**Figure 3:**
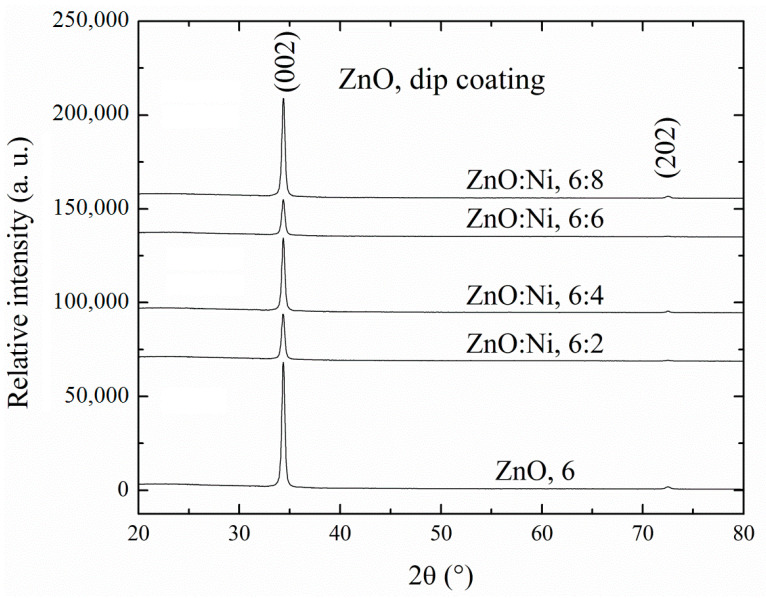
Shows the X-ray diffraction patterns of undoped ZnO films and doped ZnO films with different dippings in the nickel solution, both deposited by dip coating.

**Figure 4:**
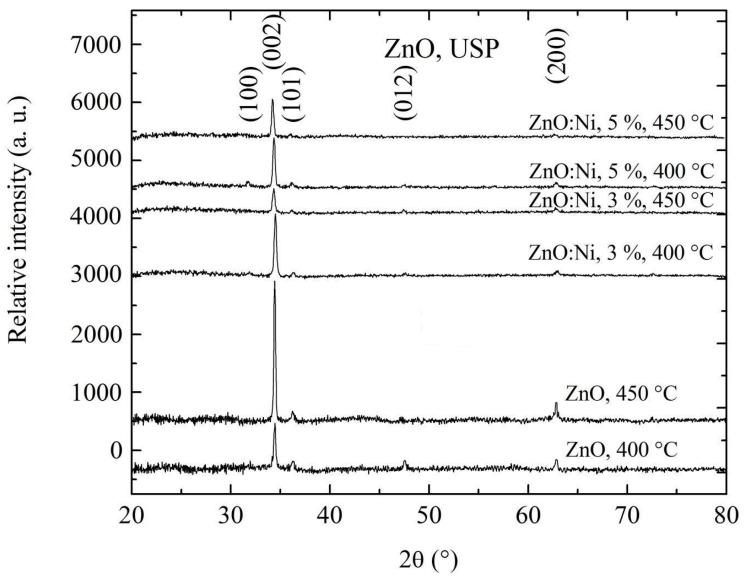
Shows the X-ray diffraction patterns for undoped ZnO films and doped ZnO:Ni films deposited with different Ni concentrations and at 400 and 450 °C, both prepared by USP.

Figure 3 shows the X-ray diffraction spectra of ZnO films obtained by dip coating. All the measured peaks coincide with that of JCPDS card no. 36–1451 for wurtzite type ZnO structure [61]. In Figure 3 the diffraction spectra for the ZnO films deposited by dip coating are shown. Some spectra, corresponding to the ZnO:Ni, 6:6, Figure 3 or ZnO:Ni, 3%, 450 °C, Figure 4 films, show less intensity in the (002) preferential plane in comparison to the other spectra, due to the grain growth along diverse directions, thus affecting the crystalline quality, and hence less reflection of (002) planes. The X-ray spectra for ZnO films deposited by USP are shown in Figure 4, which contains similar preferential orientation (002) as the ones deposited by dip coating. However, the diffraction peaks of the USP deposited films are less intense and more numerous due to less crystalline. A much-detailed analysis of these two spectrums are presented as follows.

The average crystallite size, *D*, and the lattice constants, *c*, and *a* of the ZnO films deposited by both the deposition techniques were estimated from the X-ray spectra [62]. D was deduced from the (002) peak, using the Scherrer’s formula, as shown in Equation (6) [63]:

(6) $$D=\frac{0.89\lambda }{\beta \cos\theta }$$

where λ is the wavelength of X-Ray (λ = 1.5405 Å), *θ* the diffraction angle and *β* the full width of the diffraction line measured at half of its maximum intensity, FWHM.

The lattice constants, *c* and *a* can be estimated by using (002) and (101) planes, according to the Equations (7) and (8) [64].

(7) $$d_{\mathrm{hkl}}=\frac{\lambda }{2{\sin\theta }_{\mathrm{hkl}}}$$

(8) $$d_{\mathrm{hkl}}=\frac{1}{\sqrt{\left[4\left(h^{2}+\mathrm{hk}+k^{2}\right)/3a^{2}\right]+l^{2}/c^{2}}}.$$

whereas d_hkl_ is inter planar distances along the crystallographic directions (002) and (103), obtained from the Bragg’s law, and θ_hkl_ is the diffraction angles of the (002) and (103) peaks.

The texture coefficient (T_C_(hkl)) is the crystalline orientation of a particular plane, T_C_(hkl), it is obtained, through the calculation of the magnitudes of the peaks l(hkl), and using the next relation [65].

(9) $$T_{C}\left(\mathrm{hkl}\right)=\frac{l\left(\mathrm{hkl}\right)/l_{s}\left(\mathrm{hkl}\right)}{n^{-1}\sum_{n}l\left(\mathrm{hkl}\right)/l_{s}\left(\mathrm{hkl}\right)}$$

where n is the number of peaks, l(hkl) and I_S_(hkl) are the measured relative intensity of planes to the (hkl) direction and standard intensity of planes to the (hkl) direction taken from JCPDS datebase, respectively. For T_C_(hkl) values higher than 1, mean that there are a lot of planes oriented to the (hkl) direction, and for T_C_(hkl) values equal to or less than 1, mean the deposited material that shows randomly oriented planes.

An estimate of the dislocation density was also obtained: *δ*, which is the amount of defects in the film and it is defined as the length of dislocation lines per unit volume of the crystal, to obtain *δ*, the Equation of Khan was used [66].

(10) $$\delta =\frac{1}{D^{2}}$$

where *D* is average crystallite size. The strain, *ε_zz_* of the films is obtained from the c-axis lattice parameter using the equation de Ong [67].

(11) $$\varepsilon_{zz}=\frac{c-c_{o}}{c_{o}}\times 100\%$$

where *c* is the lattice constant, which is obtained from spectra of X ray, Figure 3 and Figure 4, and *c_o_* = 5.20 Å is the unstrained lattice constant of ZnO based on the Equation (8), if *ε_zz_* has positive values, this show tensile strain while negative values represent compressive strain.

The Table 3 shows the average crystallite size, *D*, the lattice constants (*c* and *a*), T_C_(002) and thickness film in ZnO films deposited by both deposition methods. ZnO has lattice constants *a_o_* = 3.25 Å and *c_o_* = 5.20 Å [68]. For ZnO thin films deposited by dip coating, the values of the lattice constants and average crystallite size were calculated to be approximately 5.21 Å and 35 nm, respectively, irrespective of the dippings in the Ni solution or variation in the Ni concentration, Section 3.3, lattice constant *a* could not be obtained because it does not appear the (103) peak in these spectra. It can be observed that the number of dippings into the Ni solution does not modify the structural properties significantly. However, for films deposited by USP, increase in the depositing temperature resulted in higher crystallite size. The authors believe that the smaller crystallite size of doped ZnO films could be due to the presence of Ni ions inside its crystal structure and also for doped ZnO films deposited with 3 at. % and 400 °C, a large amount of Ni ions may be allocated in the grain borders occupying interstitial places, inhibiting the growth of crystallites [69]. However, as the depositing temperature is increased, authors believe that the Ni ions can be distributed occupying the substitutional places, allowing the crystal to grow [69,70]. Furthermore, an increase in the Ni in solution resulted in the elongation of *a* lattice constant and an compression of *c* lattice constant due to the difference in the ionic radii of Zn^2+^ and Ni^2+^, and the depositing temperature results in an effect that is contrary over the lattice constants, this is, elongation of *c* and compression of *a*. Regarding the texture coefficient, T_C_(002) for ZnO films deposited by dip coating, it is observed that when the number of dippings in the Ni solution is increased, it helps to rise the T_C_(002) up to 6 dippings in which T_C_(002) decreases slightly and it is kept constant. For undoped ZnO films deposited by USP, the depositing temperature helps to have a higher number of crystalline planes to (002) direction. On the other hand, for doped ZnO films, there was an increase in the depositing temperature decreased the T_C_(002), which is in accordance with the increased crystallite size. It has been reported by Yaqin Wang et al. [71] that the increase in crystallite size results is due to the high mobility of atoms during the growth, which in turn also promotes growth in simple different planes reducing the texture coefficient, which is exactly observed in Ni doped ZnO films deposited by USP technique. Regarding the estimate of the dislocation density and strain values for ZnO deposited by dip coating, there is no variation with respect to the number of dippings or the impurity concentrations; however, for undoped and doped ZnO films deposited by UPS, the dislocation density increases as doping in a starting solution increases and decreases as the substrate temperature increases. Regarding the strain, the lattice constant, *c* expands by the presence of the dopant in the crystal lattice and, for ZnO films deposited by USP, lattice constant, contracts reaching its value in bulk, *c_o_* when the substrate temperature is increased. On the other hand, the dislocation density decreases as the substrate temperature is increased for ZnO films deposited by UPS, and it increases with the increment of the incorporation of dopants in the crystal lattice. For the effects of comparison, the dislocation density is calculated in the (002) crystallographic direction.

While the thickness of the films deposited by USP is very slightly affected by the substrate temperature, the films deposited by dip coating showed an increase in thickness of approximately 15 to 20 nm, with each deposited layer as shown in Table 3.

### 3.3. Morphological Properties for ZnO Films Deposited by Dip Coating and USP

**Figure 5:**
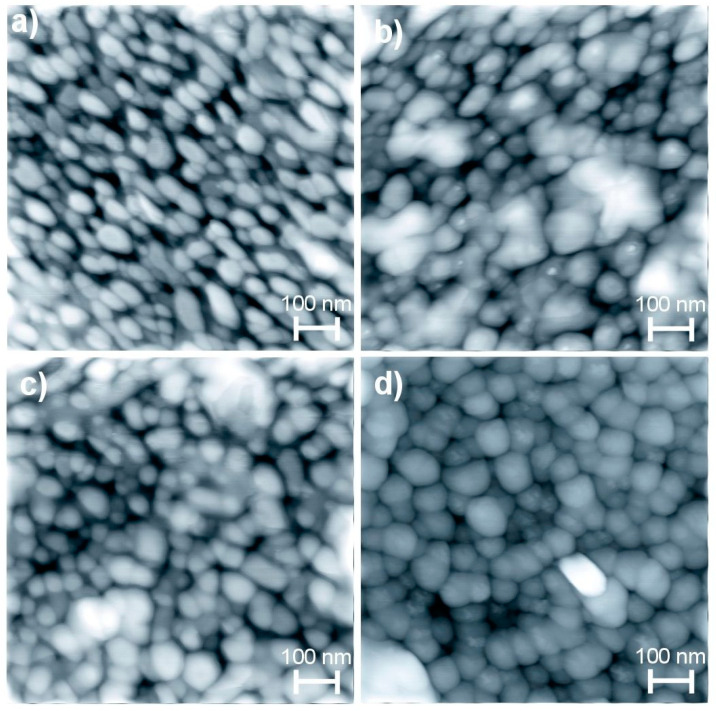
SEM micrographs of ZnO films prepared by (**a**) zero, (**b**) two, (**c**) four and (**d**) eight dipping’s in the Ni solution.

Figure 5a–d show the SEM images of undoped and doped ZnO films deposited by dip coating method with zero, two, four and eight dipping’s in the Ni solution, respectively. In general, the surface morphology of all ZnO films was granular and porous, with a wide grain size distribution. The range of the grain sizes was obtained from the image processing techniques. Figure 5a–c show surfaces formed by grains with elongated circles-shaped geometry, large holes at regular intervals and an average grain size ranging between 40 and 150 nm in diameter. However, Figure 5d shows a more compact surface (less porous surfaces) and bigger grains of approximately 100–150 nm in size, and the surface is formed by spherical grains, which are interconnected forming linked chains, in the form of agglomerates.

**Figure 6:**
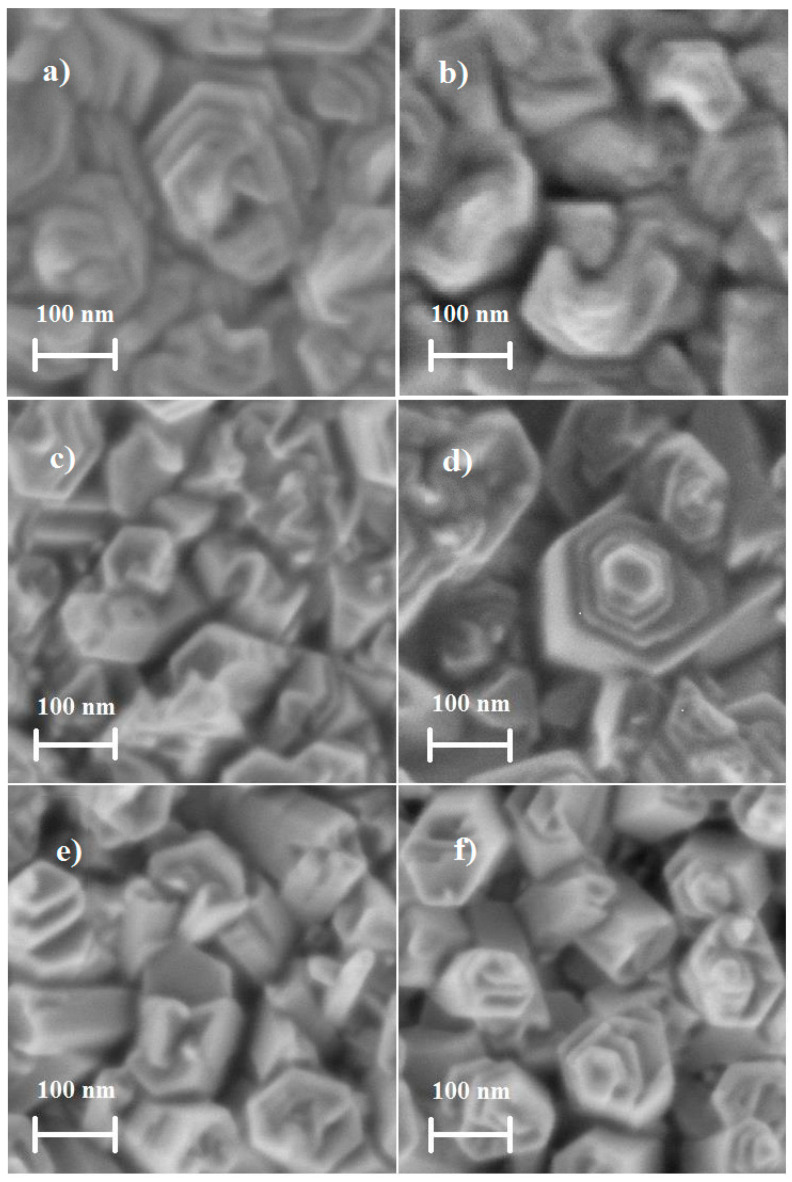
SEM images of undoped ZnO films deposited at (**a**) 400 °C, (**b**) 450 °C, doped ZnO films with 3 at. % and at (**c**) 400 °C, (**d**) 450 °C, and with 5 at. % at (**e**) 400 °C, and (**f**) 450 °C.

SEM images of the undoped and doped ZnO films deposited by USP are shown in Figure 6a–f. The films present a similar aspect, that is, grains tend to form non-definite geometric structures, possibly resembling tripods, pyramids, hexagonal and rose-petals, the grain size of which varies from 90 to 250 nm as shown in Figure 6a–f, although the large grains may be due to the result of the agglomeration of smaller grains, as reported by A. Smith et al [72].

The Ni concentration does not favor the growth of the grains as shown in Figure 6c–f. Hence, it can be concluded that the Ni concentration has a very little effect on the crystal size as well as the grain size of the doped ZnO films. The preferential direction or the amount of these crystalline planes oriented towards some crystallographic direction depends on the deposition techniques and the depositing conditions. If the sample represents just one preferential crystal orientation, the surface morphology is usually formed by just one grain shape and these are large, (the growth of the films is preferably taken in the direction [0001], due to fact that the system tends to stabilize decreasing the surface energy) just as it is with the surface morphology of the ZnO films deposited by dip coating, Figure 5. In another way, if the crystal structure shows up with different crystalline orientations, the surface morphology will be formed by grain growth in different orientations, shapes and sizes, just as the morphology of the films deposited by UPS as seen in Figure 6. An important factor in the crystallinity [73], growth rate [74], morphology [75] and, in general, the structural properties is the distribution of thermal energy during the film growth.

### 3.4. Secondary Ion Mass Spectrometry Measurements

**Figure 7:**
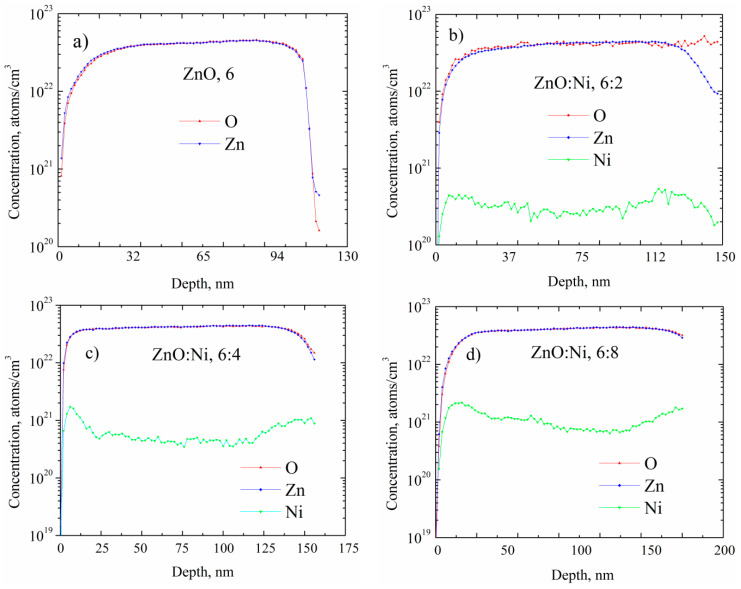
SIMS depth profiles for ZnO films doped with (**a**) 0, (**b**) 2, (**c**) 4 and (**d**) 8 dippings in Ni solutions.

SIMS (Secondary Ion Mass Spectrometry) depth profiles were plotted in order to obtain the distribution and the amount of the elements into the ZnO films [76], which is crucial to know the amount of the Zn, O and Ni in the ZnO due to the direct influence in the sensing properties. Figure 7 shows SIMS profiles of undoped and Ni doped ZnO films deposited by dip coating. It is observed that Ni atoms were incorporated significatively at 15 nm in depth. This is due to the diffusion of the Ni ions, molecules, cumulus or particles (it is unknown how the Ni is incorporated and the exact spot where it is found into the crystal lattice) through the porous regions into the ZnO film constituted by layers previously deposited, which is further assisted by the thermal processing. O and Zn concentrations in all the SIMS profiles are similar about 4.2 × 10^22^ atoms/cm^3^ while the average Ni concentration shows an increasing trend with the increase in the number of dippings in Ni solutions as shown in Table 4. The concentration of the elements Zn, O, Ni was obtained from the mean value in the volume. As mentioned in Section 2.1.2, six dippings in the zinc acetate dehydrate solution were necessary to form the ZnO layer and 2, 4, 6 and 8 dippings in a nickel acetylacetonate solution to dope the ZnO film. From the X-ray results it was observed that an extra layer of NiOx or any other layer was not deposited. The dip coating technique is very efficient to incorporate atoms, and in the optimal use of chemical solutions, wasting very little material compared to the spray pyrolysis technique. It is also observed that, higher is the number of dippings, the greater is the incorporation of Ni atoms in the ZnO films, until reaching their maximum solubility limit in the ZnO. However, at higher dippings, more Ni atoms continue to be incorporated, although in the form of conglomerates mainly at the grain boundaries.

**Figure 8:**
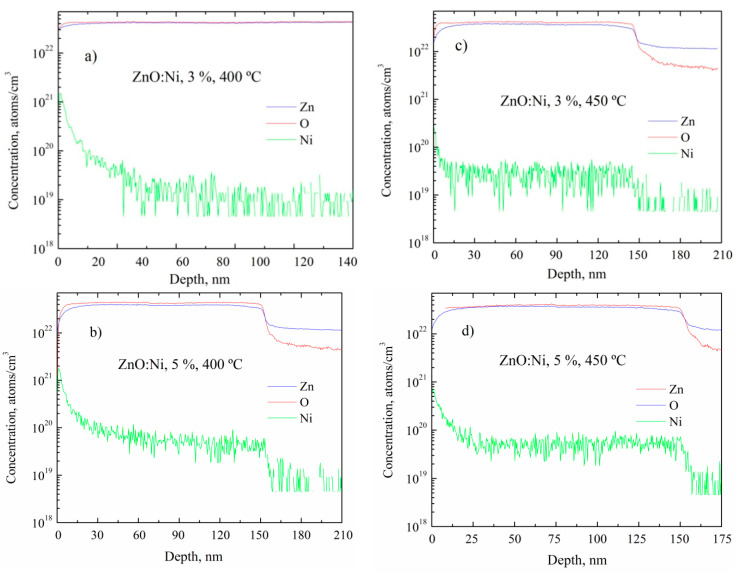
SIMS depth profiles for ZnO films doped with 3 at. %, at (**a**) 400 °C and (**b**) 450 °C and for ZnO films doped with 5 at. %, at (**c**) 400 °C, and (**d**) 450 °C.

In Figure 8, the SIMS depth profiles are shown for 3 and 5 at. % doped ZnO films prepared by USP, and deposited at 400 and 450 °C. The Ni depth profiles are constant across the bulk and the amount of Ni observed for ZnO films prepared by USP, is relatively lower compared to that deposited by dip coating as shown in Table 4. In the sensing process, the Ni atoms on the surface, favor the adsorption of oxygen and hence elevate the sensing response. Apparently, the amount of Ni atoms at the surface of films prepared by both depositing techniques is similar, although it is believed that there are more Ni atoms on the surface for the ZnO films deposited by dip coating, as shown in Figure 7 and Figure 8. Table 4 presents the Ni concentration in bulk for ZnO films deposited by both deposition techniques. It is evident the ZnO films deposited by dip coating have more Ni atoms in bulk (10 orders of magnitude) than ZnO film deposited by USP. Therefore, it can be affirmed that the dip coating method favors greater Ni incorporation into the crystal lattice. Therefore, the crystalline quality helps to incorporate impurities into the crystal lattice, as shown in Table 3 and Table 4. On the other hand, the ratio of zinc and oxygen atoms are similar in bulk for all deposited ZnO films; however, depending on the depositing condition it proportion changes on the surface, giving a non-stoichiometry character, it is believed that there are more oxygen atoms than zinc atoms, which is important for the sensing properties.

### 3.5. Sensing Properties

The sensing performance of both undoped ZnO and Ni-doped ZnO films deposited with dip coating and USP as a function of the C_3_H_8_ and CO concentration and operating temperature are presented and compared. The ratio error in measurements performed is around 5%. This ratio error was obtained for one measurement, five measurements were made at different sites of the sample and an average was taken; an error bar was put on every measurement.

#### 3.5.1. Selectivity

An important parameter that determines the sensor performance is selectivity. In this work, two gases (CO and C_3_H_8_) were tested for all the ZnO samples obtained in this work. 

In general, all ZnO films show a surface resistance change at operating temperatures above 100 °C, since the thermal energy provided by the heater, is enough for release the molecular oxygen adsorbed on the surface and bound to the gas molecules. At higher C_3_H_8_ concentrations than 300 ppm, sensing response occurs for some doped ZnO thin films, because more surface oxygen stays on the surface for additional desorption.

**Figure 9:**
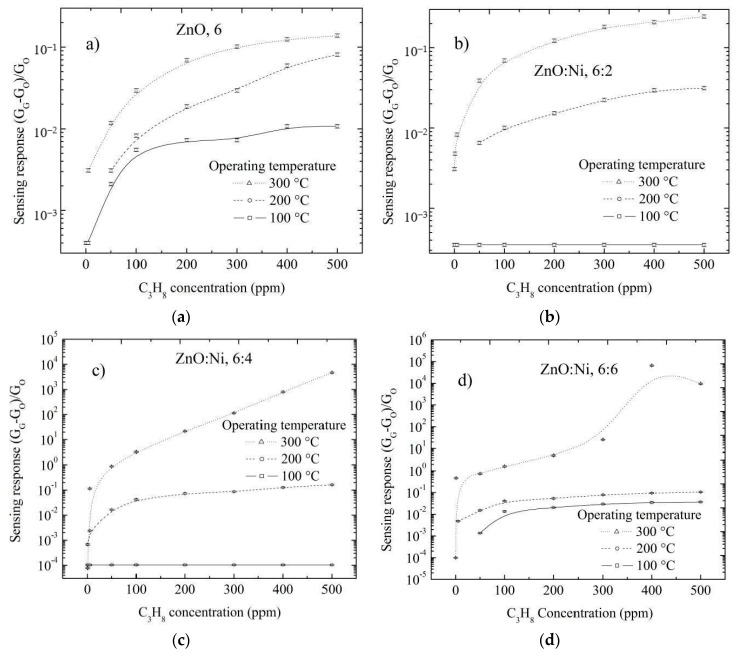

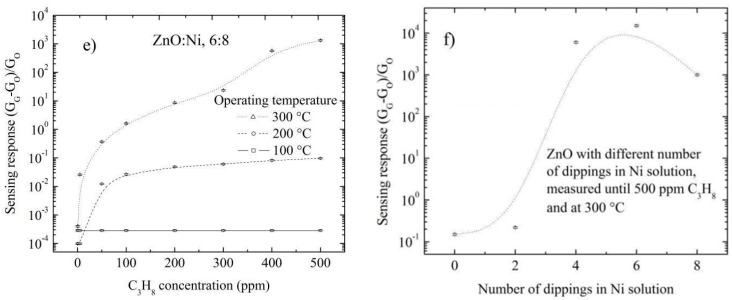
Sensing response depending on the propane concentration for ZnO films doped with different number of dippings: (**a**) zero, (**b**) two, (**c**) four, (**d**) six and (**e**) eight; and measured at 100, 200 and 300 °C, (**f**) maximum sensing responses registered for ZnO films measured with 500 ppm and at 300 °C.

Undoped ZnO and doped ZnO films deposited by dip-coating are then analyzed in an C_3_H_8_ atmosphere. The sensing response values at different number of dippings in the Ni solution are shown in Figure 9a–e. As the number of dippings increases, the variation in the surface electrical resistance of ZnO films increases, which makes Ni a good catalyst for enhancing gas sensing.

Maximum gas sensing response was obtained for Ni doped ZnO films doped with 6 dippings, as is shown in Figure 9d; further increase in the number of dippings did not cause any significant change in surface electrical resistance according to Figure 9e. This can be attributed to the fact that ZnO surface was saturated with Ni atoms in the form of agglomerates, which subsequently prevents additional adsorption of oxygen. Maximum sensing responses were registered at 500 ppm of C_3_H_8_, and measured at 300 °C as depicted in Figure 9f.

**Figure 10:**
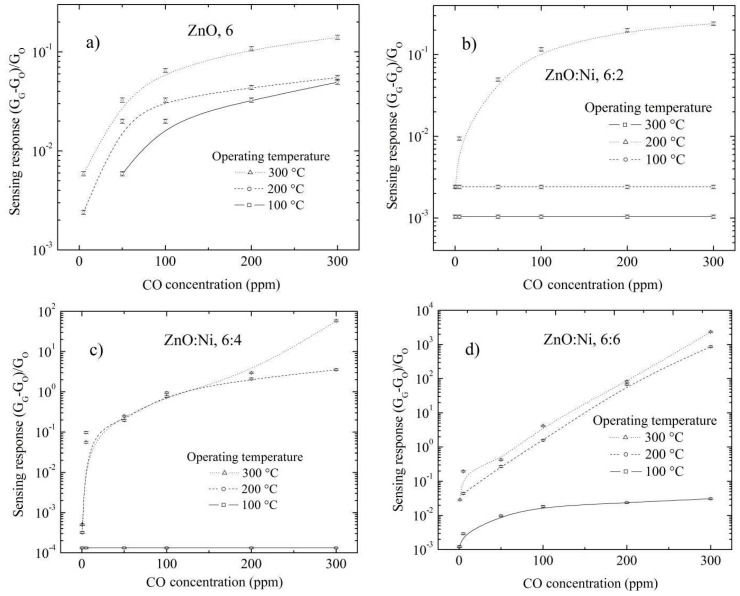

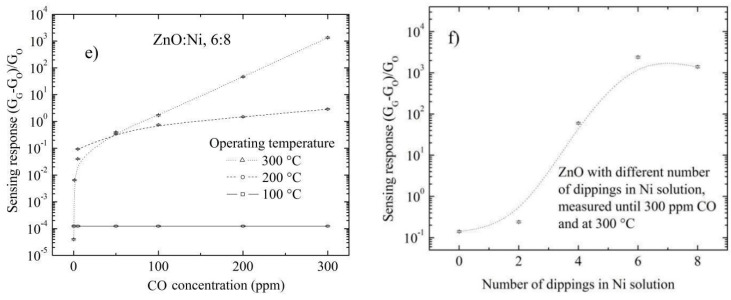
Sensing response depending on the carbon monoxide concentration for ZnO films doped with different number of dippings: (**a**) zero, (**b**) two, (**c**) four, (**d**) six and (**e**) eight; and measured at 100, 200 and 300 °C, (**f**) Maximum sensing responses registered for ZnO films measured with 500 ppm and at 300 °C.

The sensing properties for undoped ZnO and doped ZnO films deposited by dip-coating and with different number of dippings in the Ni solution and in an environment of CO are presented in Figure 10a–e. The results of sensing properties in a CO environment are similar to the ones in a C_3_H_8_ environment, with the only difference that the values of the sensing responses are lower for the case in the CO environment; due to the C_3_H_8_ molecules approaching the hot sample, this is broken into different fractions with the possibility of reacting with the surface; in this way the C_3_H_8_ molecules react with the surface more than with the CO molecules.

**Figure 11:**
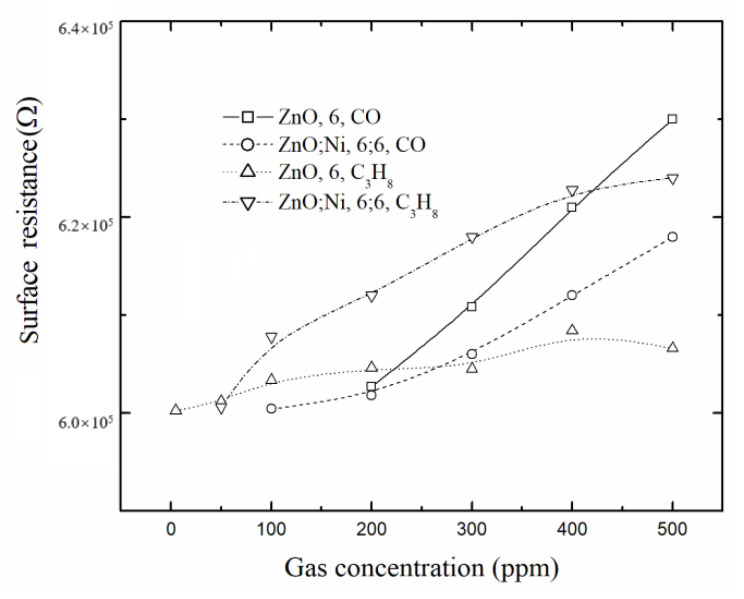
Surface electrical resistance as a function of the gas concentration for ZnO films deposited by dip coating at various conditions.

In Figure 11, the surface electrical resistance values are presented as a function of the gas concentration for the ZnO films deposited by dip coating and measured at 100 °C and at different gas concentrations, in which it can be observed that even at low temperature, there is a change in the surface electrical resistance.

**Figure 12:**
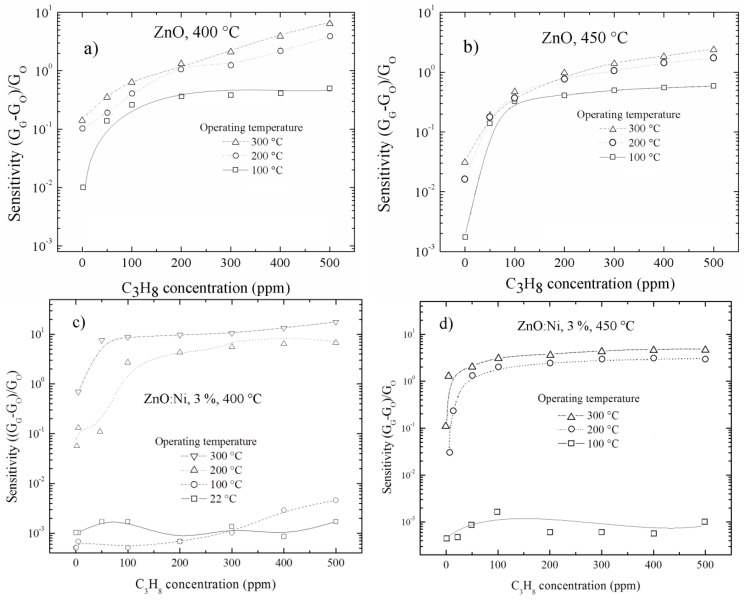

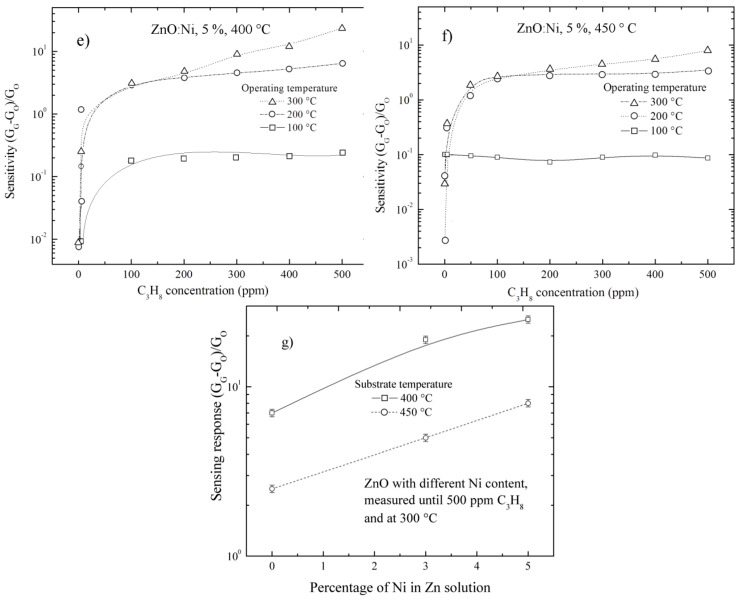
Sensing response as a function of C_3_H_8_ concentration for undoped ZnO films at (**a**) 400 °C, (**b**) 450 °C and doped with different percentages of Ni content (**c**), 3 at. %, 40 °C, (**d**) 3 at. %, 450 °C, (**e**) 5 at. %, 400 °C, (**f**) 5 at. %, 450 °C, (**g**) Maximum sensing responses for ZnO films doped with different percentages of Ni content and at two substrate temperatures.

The C_3_H_8_ sensing properties for undoped ZnO and doped ZnO films deposited by USP are analyzed below. Figure 12a–f show the sensing response of films deposited with different deposition conditions, propane concentrations and at operating temperatures. It is observed that the sensing response is increased as operating temperature increases, and this reaches a sensing response of around 6 for undoped ZnO films prepared at 400 °Cand measured with a propane concentration of 500 ppm and at 300 °C, Figure 12a.

The sensing response presents low values for low C_3_H_8_ concentrations and at operating temperature below than 300 °C. The sensing responses are low at 100 °C, increases notoriously till 200 °C and it is increases to its maximum value at 300 °C, which apparently is the best operating temperature to have high response. The maximum sensing responses are present at 500 ppm and operating temperature of 300 °C, and these are shown in Figure 12g. This behavior is similar to what was explained for ZnO films prepared by dip coating, i.e., the necessity of more thermal energy to produce more adsorption–desorption reactions on the surface. The sensing response at different propane concentrations in undoped ZnO films, prepared at 450 °C is similar as mentioned above in this section; however, it turns out to be smaller compared to films prepared at 400 °C, which can be clearly seen in Figure 12b. This might be due to the increase in the quality of the films as the substrate temperature increases, thus decreasing the structural defects, since it is decreasing the dislocation density, strain and increase in the crystallite size *D* (see Table 3), and therefore the dangling bonds, which is what causes the lowering of the oxygen adsorption.

For ZnO films doped with 3 and 5 at. % and deposited at 400 °C, the sensing response behavior is similar to the undoped ZnO films for different C_3_H_8_ concentrations. The sensing response for ZnO films doped with 3 at. % is relatively higher than undoped ZnO films, as shown in Figure 12c. This is due to the presence of Ni atoms on the surface, which acts as a catalyst increasing the adsorption of oxygen; however, for ZnO films doped with 5 at. %, the sensing response continues to increase, which is dissimilar to films deposited by dip coating with eight dippings as displayed in Figure 10e. For doped ZnO films deposited at 450 °C, the behavior of sensing responses was similar, but with low values compared to the films deposited at 400 °C as evident in Figure 11f. The explanation is the same as the one of the undoped ZnO films.

**Figure 13:**
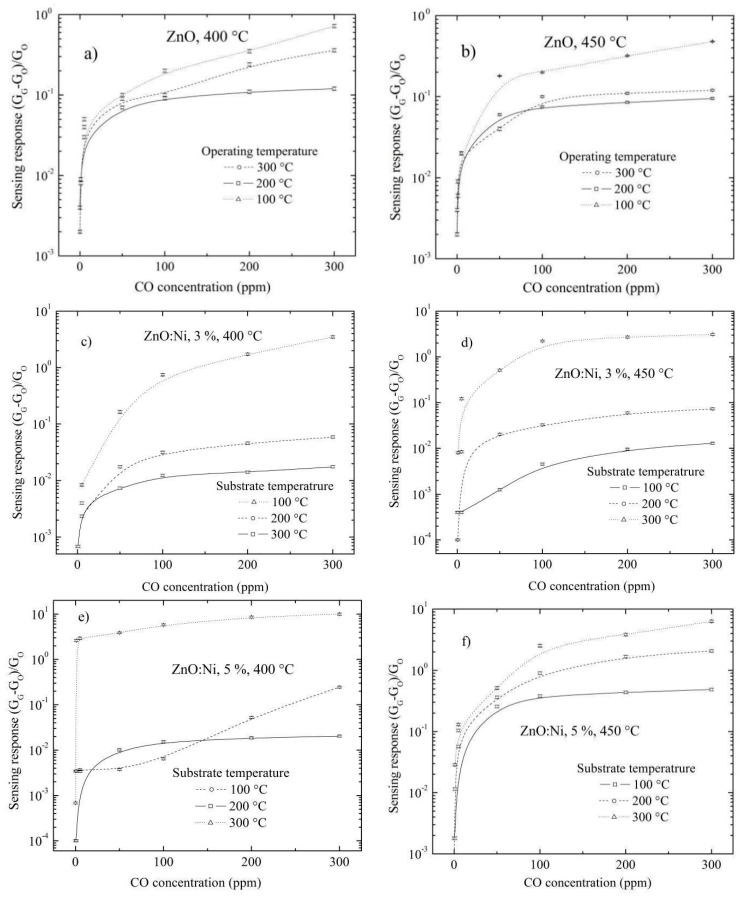

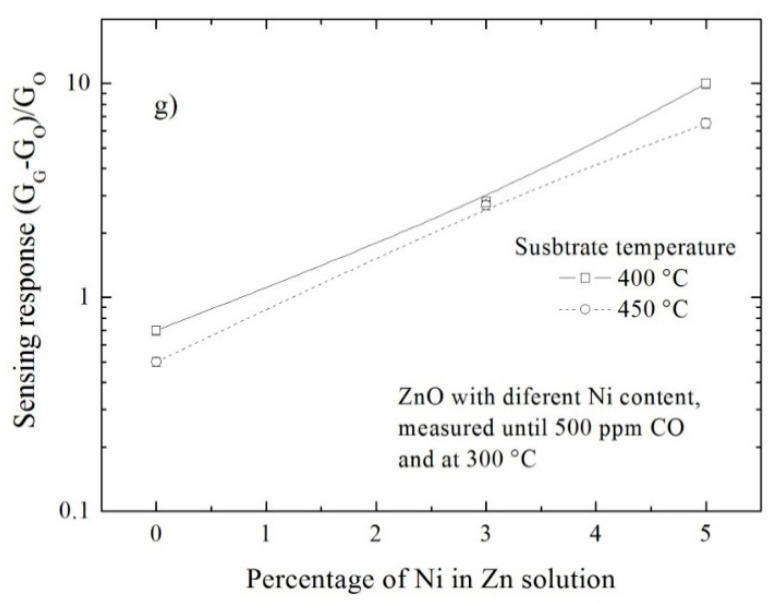
Sensing response as a function of CO concentration for undoped ZnO films at (**a**) 400 °C, (**b**) 450 °C and doped with different percentages of Ni content and substrate temperature (**c**) 3 at. %, 400 °C, (**d**) 3 at. %, 450 °C, (**e**) 5 at. %, 400 °C, (**f**) 5 at. %, 450 °C, (**g**) Maximum sensing responses for ZnO films doped with different percentages of Ni content and at two substrate temperature.

The results of sensing responses for undoped ZnO and doped ZnO films deposited by UPS and in an environment of CO are present in Figure 13a–f. In the same case, the sensing responses of ZnO films deposited by dip coating in a CO environment were smaller compared to ZnO films in a C_3_H_8_ environment. The heath that the heather radiates causes the C_3_H_8_ molecules to become fragmented, causing a higher possibility of reaction of the C_3_H_8_ molecules with the ZnO surface; hence, in general, the sensing responses are higher in a propane environment than in carbon monoxide. Furthermore, the sensing responses decrease as the number of dippings in Ni solution increases, from the eighth dipping for the ZnO films deposited by dip coating in both a C_3_H_8_ and CO environment (Figure 9 and Figure 10); however, the sensing response increases as the Ni in Zn solution increases for the ZnO films deposited by UPS in both a C_3_H_8_ and CO environment (Figure 12 and Figure 13). In the next section, we analyze the sensing responses in the function of the Ni concentration in crystal lattice.

#### 3.5.2. Doping Concentration Dependence CO and Propane Response

**Figure 14:**
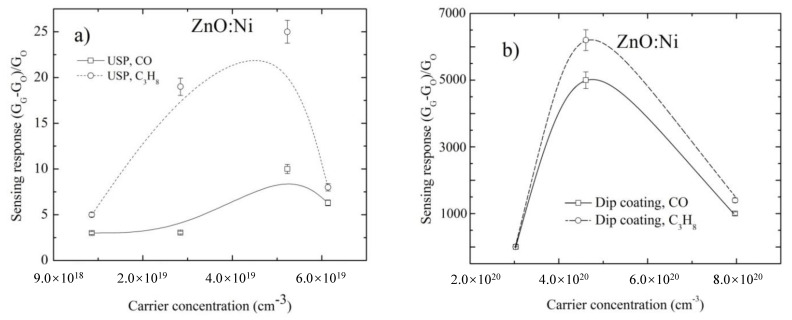
Maximum sensing response as a function of Ni concentration inside the crystal lattice for all ZnO films deposited by USP (**a**) and dip coating (**b**), measured with CO and C_3_H_8_ gases at 300 ppm and 300 °C.

All maximum sensing responses as a function of Ni concentration in ZnO films are presented in Figure 14. These results for ZnO films were obtained by employing the two deposition techniques and ambient gases at 300 ppm and 300 °C. It was observed that all sensing responses increase as the Ni concentration increases for all ZnO films deposited by any deposition technique and in the presence of any gas, which confirms the effectiveness of the Ni as catalyst. Therefore, in order to have a higher sensing response, greater incorporation of Ni is necessary into the substitutional sites mainly located on the surface; these substitutional sites are energetic states that function as oxygen traps. Oxygen is the intermediate atom that regulates the surface electrical resistance, since it is the cause of capturing free electrons. When this oxygen is dislodged from the surface either by the presence of a gas or by operating temperature, it releases these captured electrons. To have a high concentration of surface energy states available and thus have high Ni concentration, a large effective surface area is necessary. Furthermore, a high crystal quality increases the concentration of surface energy state. From the results of the X ray (Table 3), it was observed that the films with the best crystalline qualities, i.e., the lattice constants *a* and *c*, are close to those of volume, show large crystal size *D* and texture coefficient T_C_(hkl) and therefore show high sensing responses such as the ZnO films deposited by dip coating.

On the other hand, the ZnO films deposited by USP have low incorporation of Ni into their crystal lattice, which can be assigned to its fast deposition process, thus limiting its crystal quality (there is not enough time for the atoms to place themselves in an orderly way), which shows a lower sensing response.

Now, at a certain value of the Ni concentration, above which the sensing response drops (as can be observed in the Figure 9f and Figure 10f), a certain fraction of Ni atoms is not accommodated in substitutional sites, but instead incorporate themselves in the grain boundaries forming agglomerations, thus inhibiting the sensing response.

From Figure 14, it is clear that the best sample for detecting CO and C_3_H_8_ are doped ZnO sensors obtained by Dip coating method. The obtained sensing responses by dip coating method are higher in two orders of magnitude in comparison with the sensors obtained by the USP method.

#### 3.5.3. Effect of Surface Morphology on the Sensing Response

It is a common observation that morphological properties play an important role in determining the quality of the sensing process for undoped ZnO films. In our previous work, a qualitative model for gas sensors was proposed, which considers the sensing response to be a function of the surface morphology, i.e., size, shape and separation between grains, among others [77].

It was mentioned earlier that a surface morphology, composed of small and irregular grains and with secondary grains formed by smaller crystallites separated by a minuscule space in between, results in an increase in grain boundaries and hence the potential barriers, which enhances the sensing response of the semiconductor oxide in thin film form.

Therefore, the ZnO films deposited by USP exhibit a surface buildup of irregular and hexagonal shaped grains (Figure 6), which favors a better attachment than the films deposited by dip coating with surface formed of round grains (Figure 4). As a result, the films deposited by USP are the most appropriate ones for gas sensing. In Table 5, the grain shape, grain size, volume occupied by the grains or Vol_Grain_/Vol_Empty_ or porosity and effective surface area obtained by image processing techniques and the height of the grains obtained by profilometer measurements are presented. The image processing techniques are based on distance values given to each pixel constituting the digitized image. The 3-D images give a value of distance to each pixel in the x or y axis and in the z axis a value in the grayscale. The clear pixels represent a high region and the dark pixels represent low regions, the addition of which represents the total area or effective surface area. From Table 5, the measurements presented were made on samples with flat surfaces of 0.25 μm^2^.

From the sensing response and surface morphology and Ni concentration results presented, we can observe four things: (a) For the undoped ZnO films, the highest sensing responses correspond to the ones deposited by USP, which is due to the fact that they have more adequate surfaces formed of small, irregular grains with good coupling, and higher effective superficial area; (b) For the doped ZnO films, the higher sensing responses correspond to the films prepared by dip coating, due to efficient incorporation of Ni impurities into the ZnO crystal lattice as evident from Table 4 and Figure 14, which is due to a higher crystalline quality, which allows for a better incorporation of the Ni in substitutional sites and, in turn, these Ni impurities favor the adsorption of oxygen molecules. The dip coating method consists of depositing one layer on top of another, the first layers are undoped ZnO and on the top are the doped layers, but it is the final annealing process, which predominantly governs the uniform distribution of impurities across the film. The dip coating technique is a process facilitating a better control of the growth parameters than the USP technique, and so deposits film with better structural properties, along with a higher proportion of the Ni impurities that stay on the surface occupying substitution sites; (c) Undoped and doped ZnO films deposited by USP at a substrate temperature of 450 °C have less sensing response than the films deposited at 400 °C, although the ZnO films deposited at 450 °C have more Ni concentration; this is because the films deposited at 400 °C have amore adequate surface morphology to detect gases (Figure 12g and Figure 13g), which is due to the fact that its surface is constituted by a greater amount of small grains and with a good coupling in between them (Table 5), creating larger quantity of grain borders; (d) From Figure 14, it is observed that a certain quantity of the Ni impurities incorporated into the ZnO crystal lattice, which makes the sensing response drop for the films deposited by both methods, which is because the Ni atoms are located mainly in the inter-grain regions, forming conglomerates or clusters, which subsequently invalidates the potential barriers and prevents the additional adsorption of oxygen molecules from the atmosphere. However, it is observed that a smaller amount of Ni concentration is required to inhibit the potential barriers in the case of films deposited by USP (Figure 14); this may be due to the fact that their size of the inter-grain regions are smaller compared to films deposited by dip coating (Figure 5 and Figure 6); more research is needed.

#### 3.5.4. Temperature Dependence CO and Propane Sensing Response

Temperature has pronounced effects on ZnO sensing responses, as it influences not only the physical properties of semiconductors (change of the free carrier concentration, Debye length, etc.), but also it determines every reaction taking place at the surface, as well as the most probable species adsorbed. In the case of gas sensing application, we are concerned about the temperature effect on the properties related to the processes occurring at the surface of the sensor. Adsorption and desorption processes are not only temperature activated processes, but also depend on the surface coverage by molecular and ionic species, nature of chemical decomposition and the presence of reactive sites.

The activation of the adsorption and desorption of oxygen at the semiconductor surface requires temperature. In other words, the higher the operating temperature, the greater the activation processes on the surface of the ZnO semiconductor. With its high electron affinity, adsorbed oxygen traps free electrons on the semiconductor, forming a potential barrier over the surface. The electrons on their way, pass through the grain boundaries, overcoming the potential barrier which control the electron flow. As we mentioned in Equation (2), the height of the potential barrier depends on doping concentration, N_D_ and on surface states density, N_SS_, which in turn depends on the amount of impurities that occupy substitutional states on the surface.

In our results, it was observed that, in order to have a sensing response, a minimum of 100 °C was required. Apparently, at operating temperatures higher than 300 °C, there will be a greater sensing response in the ZnO films deposited by dip coating than in the films deposited by spray pyrolysis, because of a greater amount of surface states, N_SS_.

#### 3.5.5. Transient Resistance at Optimum Doping Concentration for CO and Propane Gas

The transient resistance is the surface electrical resistance measured on the multimeter using two points, as described in Section 2.2. Table 6 shows the values of the transient resistance given in MΩ, for samples that showed the highest sensing responses with the appropriate measurement conditions, that influences them. It is observed that at operating temperature of 300 °C and without the presence of gas, the transient resistance is close to 300 MΩ. Furthermore, the values of this electrical resistance are lower in the presence of gas at concentrations from 300 to 500 ppm, for ZnO films deposited by dip coating. The values of surface electrical resistance with respect to different gases and concentrations vary as there is no clear trend, but from the comments in Section 3.5.1, we believe that the C_3_H_8_ molecules approaching the hot sample are broken into different fractions, with the possibility of reacting more with the surface in comparison to the CO molecule. As a result, the electrical resistance would vary more at higher gas concentrations in the presence of C_3_H_8_.

## 4. Conclusions

Undoped and Ni doped ZnO films were prepared by the dip-coating and ultrasonic spray pyrolysis methods, utilizing similar starting precursor and doping solution. X-ray diffraction analysis show that all films have hexagonal wurtzite structure with a (002) preferential orientation along with additional peaks like (100), (101), (012) and (200) for ZnO films deposited by USP. The ZnO films deposited by dip coating have a better crystalline quality compared to the ZnO films deposited by USP because of the similarity of their lattice parameters to the material in bulk. In general, the surfaces were rough and porous. For films prepared by USP, the surface is formed by small grains with irregular geometries, perhaps resembling pyramids and hexagonal slices with rose-like structures, providing a better coupling between grains and a greater effective surface area compared to ZnO films deposited by dip coating, which have surfaces made up of large round grains with poor coupling between them and fewer grain boundaries along with the smaller effective surface area. SIMS analysis shows Ni depth profiles along the film, although it is unknown the exact site where these impurities are located; perhaps most of the Ni atoms are in substitution sites. The ZnO films deposited by dip coating have a better incorporation of Ni atoms in substitutional sites because the nature of the depositing technique produces films of good crystalline quality, due to a slow growth process and with a good use of the chemical reactives. The present work shows that the incorporation of the Ni impurities in the crystal lattice and the superficial morphological play a major role in the sensing response. The sensing process in the films occurs both on their surface and on the grain boundaries, which have surface states, which may be loose bonds or states linked to Ni atoms that capture ambient oxygen. Undoped ZnO films deposited by USP, due to their more suitable morphology, have a greater amount of effective surface area and grain boundaries, thus favoring the detection of gases, in comparison to the undoped ZnO films deposited by dip coating. However, the doped ZnO films deposited by dip coating, possesses a greater amount of surface states occupied by Ni atoms located in substitutional sites due to their better crystalline quality than the ZnO films deposited by USP, which enhances gas detection. For both ZnO films deposited, the C_3_H_8_ gas had a better sensing response than CO gas, since the C_3_H_8_ molecules are more complex than CO molecules; therefore, when the C_3_H_8_ molecules approaches the hot sample, are broken into different fractions, with the possibility of reacting more with the surface in comparison to the CO molecules. At high Ni concentrations, the sensing response decreases, which can be due to the excess of Ni atoms located in inter-grain spaces forming clusters and inhibiting these zones, for the occurrence of any chemical reactions with atmospheric oxygen. These clusters are made of metallic Ni or other forms of ZnO and are too small to be detected by X-ray. However, they show an unfavorable effect on gas sensing response.

## Figures and Tables

**Table 1:** Comparison between our main results and that of the references.

Sensor Material	Synthesis Process	Doping Solution (at. %)	Type of Gas	Operating Temperature (°C)	Gas Concentration	Sensing Response
ZnO:Ni [21]	Sol-gel	5	Carbon oxide	350	80 (ppm)	5.5
ZnO [22]	Spray pyrolysis	---	liquid petroleum gas	377	0.4 (vol %)	32.5
ZnO:Ni	Sol-gel	---	Carbon oxide	300	300	≈3200
ZnO:Ni	Spray pyrolysis ultrasonic	5	Propane	400	500	≈25

**Table 2:** Pressure conversion table from ppm to mbar for CO and Propane.

Gas Concentration (ppm)	Carbon MonoxidePressure (mBar)	Propanepressure (mBar)
0	0.7	0.53
1	8.6	2.7
50	43	27
100	86	55
200	170	110
300	260	160
400	350	220
500	430	270

**Table 3:** Average crystallite size, D, the lattice constants c and a, texture coefficient TC(002), dislocations densities *δ*, strain *ε_zz_*, and thickness of the ZnO films deposited by dip coating and USP at different deposition conditions.

Method	Film	*D* (nm)		*c* (Å)	*a* (Å)	TC (002)		Thickness Film (nm)
	ZnO, 6	≈ 35		5.21	---	1.42		99
	ZnO:Ni, 6:2	≈ 34		5.22	---	1.43		119
Dip Coating	ZnO:Ni, 6:4	≈ 36		5.21	---	1.48		137
	ZnO:Ni, 6:6	≈ 34		5.22	---	1.45		156
	ZnO:Ni, 6:8	≈ 36		5.21	---	1.45		169
	ZnO, 400 °C	≈ 36		5.23	3.2	1.97		160
	ZnO, 450 °C	≈ 52		5.23	3.2	2.39		164
USP	ZnO:Ni, 3%, 400 °C	≈ 24		5.27	3.14	3.18		150
	ZnO:Ni, 3%, 450 °C	≈ 49		5.20	3.26	2.09		156
	ZnO:Ni, 5%, 400 °C	≈ 27		5.28	3.14	3.41		155
	ZnO:Ni, 5%, 450 °C	≈ 50		5.19	---	2.53		160
**Method**	**Film**		***δ*** **10^−4^ (nm)^−2^**				***ε_zz_*** **(%)**	
	ZnO, 6		8.1				0.19	
	ZnO:Ni, 6:2		8.6				0.32	
Dip Coating	ZnO:Ni, 6:4		8.7				0.32	
	ZnO:Ni, 6:6		8.7				0.31	
	ZnO:Ni, 6:8		8.7				0.32	
	ZnO, 400 °C		7.7				0.57	
	ZnO, 450 °C		3.7				0.57	
USP	ZnO:Ni, 3%, 400 °C		17.3				1.34	
	ZnO:Ni, 3%, 450 °C		4.1				0	
	ZnO:Ni, 5%, 400 °C		13.7				1.53	
	ZnO:Ni, 5%, 450 °C		4				0	

**Table 4:** Ni concentration for doped ZnO films deposited by dip coating and by USP.

Method	Film	Bulk Concentration (Ni atoms/cm^3^)
	ZnO:Ni 6:8	7.97 × 10^20^
Dip coating	ZnO:Ni 6:4	4.61 × 10^20^
	ZnO:Ni 6:2	3.03 × 10^20^
	ZnO:Ni, 5%, 450 °C	6.14 × 10^19^
USP	ZnO:Ni, 5%, 400 °C	5.23 × 10^19^
	ZnO:Ni, 3%, 450 °C	2.84 × 10^19^
	ZnO:Ni, 3%, 400 °C	8.57 × 10^18^

**Table 5:** Grain shape, average grain size, Vol_Grain_/Vol_Empty_ and effective surface area of the ZnO films prepared by dip coating with different dippings and that using USP with different Ni concentration in Zn solution at different substrate temperature.

Method	Depositions Conditions	Grain Shape	Average Grain Size (nm)	Volume Occupied by the Grains	Effective Surface Area (μm^2^)
	Undoped ZnO	Round	40–80	45.6	0.53
Dip Coating	ZnO:Ni 6:2	Round	60–100	48.5	0.53
	ZnO:Ni 6:4	Round	60–100	50.5	0.53
	ZnO:Ni 6:8	Round	100–150	56.9	0.54
	ZnO, 400 °C	Irregular	30–150	46.7	0.58
	ZnO, 450 °C	Irregular	40–200	49.1	0.61
USP	ZnO:Ni, 3%, 400 °C	Irregular	10–150	43.2	0.56
	ZnO:Ni, 3%, 450 °C	Irregular	10–250	44.9	0.58
	ZnO:Ni, 5%, 400 °C	Irregular	10–150	51.8	0.55
	ZnO:Ni, 5%, 450 °C	Irregular	10–150	52.1	0.56

**Table 6:** Surface resistance as a function of the gas concentration for ZnO films measured at conditions for getting optimum sensing response.

Gas Concentration (ppm)	Surface Resistance ZnO:Ni 6:6, Dip Coating C_3_H_8_, 300 °C (MΩ)	Surface resistance ZnO:Ni 6:6, Dip coating CO, 300 °C (MΩ)	Surface Resistance ZnO:Ni, 5%, 400 °C, USP C_3_H_8_, 300 °C (MΩ)	Surface Resistance ZnO:Ni, 5%, 400 °C, USP CO, 300 °C (MΩ)
0	330	334	293	290
5	225	300	291	288
10	212	295	255	62
50	189	210	133	80
100	126	60	73	59
200	54.12	3	58	35
300	12.36	0.12	26	32
400	0.005		22	
500	0.034		12	
